# Thoracic vertebral bone mineral density measured by quantitative computed tomography is associated with fracture risk in lung cancer screening populations: a prospective cohort study

**DOI:** 10.3389/fendo.2025.1672551

**Published:** 2025-11-13

**Authors:** Wenzhen Jiang, Jinchao Huang, Nan Wu, Jiaxin Liu, Jing Liang, Shixia Li, Zhaoxiang Ye, Jing Wang

**Affiliations:** 1 Health Management Center, Tianjin Medical University Cancer Institute and Hospital, National Clinical Research Center for Cancer, Tianjin, China; 2 Tianjin’s Clinical Research Center for Cancer, State Key Laboratory of Druggability Evaluation and Systematic Translational Medicine, Tianjin Key Laboratory of Digestive Cancer, Key Laboratory of Cancer Prevention and Therapy, Tianjin, China; 3 Department of Radiology, Tianjin Medical University Cancer Institute and Hospital, National Clinical Research Center for Cancer, Tianjin’s Clinical Research Center for Cancer, Tianjin, China; 4 School of Public Health, Tianjin University of Traditional Chinese Medicine, Tianjin, China

**Keywords:** quantitative computed tomography, thoracic vertebral bone mineral density, osteoporosis, computed tomography, lung cancer

## Abstract

**Background:**

Chest low-dose computed tomography (LDCT) is extensively utilized for lung cancer screening, offering a concurrent opportunity to assess thoracic vertebral bone mineral density (BMD) using quantitative computed tomography (QCT). Nonetheless, the value of thoracic BMD (TBMD) in estimating the risk of fractures within this population remains underexplored.

**Purpose:**

We sought to assess the association between fractures and QCT-based TBMD derived from chest LDCT in a lung cancer screening population.

**Materials and methods:**

A prospective study was conducted involving 546 adults aged 40 to 74 years who were enrolled in a lung cancer screening program between 2017 and 2021. TBMD and lumbar BMD (LBMD) were assessed from chest LDCT scans using QCT. Self-reported incident fractures were recorded over a 3-year period, and vertebral fractures (VFs) were evaluated on follow-up CT. Binary logistic regression models and area under the curve (AUC) analyses were utilized to develop and compare the models incorporating TBMD, LBMD, and FRAX for estimating fracture risk.

**Results:**

Out of the total participants, 323 individuals (59.2%) were found to have VFs, while 16 individuals (2.9%) reported experiencing incident fractures over a period of three years. In unadjusted logistic regression analyses, TBMD was associated with CT-detected VFs (OR = 0.955; 95% CI: 0.947 - 0.963). After adjusting for age and current smoking, TBMD remained associated with CT-detected VFs (OR = 0.953; 95% CI: 0.944 - 0.962). The optimal TBMD threshold for CT-detected VFs was 124 mg/cm^3^, with a sensitivity of 79.3%, a specificity of 70.9% and AUC of 0.823. Notably, self-reported incident fractures were significantly associated with TBMD (OR = 0.982; 95% CI: 0.965–0.999), independent of adjustment for excessive alcohol consumption (OR = 0.982; 95% CI: 0.965–0.999). The optimal TBMD threshold for self-reported incident fractures was determined to be 94 mg/cm^3^, with a sensitivity of 62.5%, a specificity of 77.0%, and an AUC of 0.678.

**Conclusion:**

QCT-based TBMD derived from LDCT scans might be a feasible and effective tool for identifying individuals with VFs and an elevated risk of incident fracture, without additional radiation exposure in lung cancer screening populations.

## Introduction

Osteoporosis is a prevalent but markedly underdiagnosed disease that increase the risk of fractures (1). Among the Chinese population aged 40 years and older, the prevalence of osteoporosis and vertebral fractures is 5.0% and 10.5% among men, 20.6% and 9.7% among women, respectively (2). Osteoporosis and its associated fractures adversely impact patients’ quality of life and contribute to an increased socio-economic burden (3, 4). However, the prevention of these fractures is feasible through timely osteoporosis screening and subsequent therapeutic interventions when necessary (5). Utilizing all available modalities could assist in narrowing the diagnostic gap.

QCT-derived BMD is a superior marker for osteoporosis screening and fracture prediction compared to DXA-derived BMD (6–8). Current clinical practice predominantly relies on LBMD (L1-L4) (9). Opportunistic QCT from non-dedicated routine CT scans allows for LBMD measurement without extra medical expenses or radiation (10–13), offering a cost-effective ancillary approach for osteoporosis screening and fracture risk assessment (14).

Chest low-dose computed tomography (LDCT) has been established as the standard clinical modality for lung cancer screening (15), providing a unique opportunity to obtain TBMD. Notably, Osteoporosis and fractures are more prevalent in lung cancer screening populations due to shared risk factors such as smoking and aging. However, the potential value of TBMD measurements derived from chest LDCT scans remains inadequately characterized, particularly regarding its capability for fractures compared to conventional LBMD measurements and the fracture risk assessment tool (FRAX). Therefore, this study sought to determine whether QCT-derived TBMD is associated with fracture risk in individuals undergoing lung cancer screening, and to compare its ability with QCT-derived LBMD and FRAX.

## Materials and methods

### Study design and participants

The data for this prospective study was derived from the Colorectal, Breast, Lung, Liver, And Stomach cancer Screening Trial (CBLAST), a multicenter, population-based study designed to investigate the effect of combined screening for the five most prevalent cancers (colorectal, breast, lung, liver, and stomach) in China. Healthy residents aged 40 to 74 years who had lived in the local community for at least three years and had no self-reported history of cancer were invited to participate in CBLAST. This study specifically included participants who completed the questionnaires and underwent baseline chest LDCT scans between June 2017 and March 2018. Participants were excluded if their T11- L2 vertebrae were not within the scanning range or if the vertebrae could not be used for BMD measurement due to lesions. The three-year follow-up assessment was completed on April 1, 2021. This study was reviewed and approved by the research ethics committee of Tianjin Medical University Cancer Institute and Hospital, and written informed consent was obtained from all participants prior to enrollment.

### Questionnaire

Data from the questionnaire were collected by trained community physicians through face-to-face interviews using touchscreen devices. The original questionnaire included variables such as age, sex, height, weight, current smoking status, alcohol consumption, previous fragility fracture, parental hip fracture, glucocorticoid use and causes of secondary osteoporosis including hyperthyroidism, type 1 diabetes, osteogenesis imperfecta, chronic liver disease, chronic malnutrition or malabsorption, and premature menopause (defined as occurring at or before 45 years of age). All incident fractures were self-reported in the 3-year follow-up questionnaires. Definitions of specific terms used in the questionnaire are detailed in **Table 1**.

**Table 1:** Definitions of specific terms.

Terms	Definitions
Current smoking	Currently smoking ≥1 cigarette per day for more than six months
Excessive alcohol consumption	Drinking ≥3 units of alcohol per day (equivalent to approximately 500 mL of beer or 100 mL of wine or 50 mL of liquor)
Glucocorticoids	Oral glucocorticoid use ≥5mg per day for more than three months
Previous fragility fracture	Fracture occurring spontaneously or arising from trauma which, in a healthy individual, would not have resulted in a fracture before baseline CT
Incident fracture	Self-reported fracture occurring during 3-year follow-up

### CT image acquisition

Participants underwent LDCT scans at Tianjin Medical University Cancer Institute and Hospital. All scans were performed using the same CT system: Definition AS (Somatom Definition AS 64, Siemens, Erlangen, Germany), with parameters consistent with those described in the study design (16). The maximum total radiation dose was 2 mSv.

### BMD evaluation and osteoporosis diagnosis

The QCT Pro system (Mindways Software, Inc., Austin, TX, USA) was used for all BMD analyses, calibrated using an asynchronous calibration phantom (Model 4). In accordance with the manufacturer’s protocols, standard QCT measurements were conducted to assess TBMD and LBMD at the T11-T12 and L1-L2 vertebrae, respectively. All measurements were performed by experienced radiologists, and no additional radiation exposure was required. The volume region of interest (ROI) was optimally positioned at the central axial level of each vertebral body (T11-L2), with a minimum cross-sectional area of 100 mm^2^ and a height of 9 mm, while excluding the basal vertebral vein, cortical bone, and sclerotic regions. Osteoporosis, osteopenia, and normal BMD were classified according to the American College of Radiology QCT diagnostic criteria of LBMD < 80 mg/cm^3^, 80 to 120 mg/cm^3^, and >120 mg/cm^3^, respectively (17).

### FRAX

The 10-year probabilities of major osteoporotic fracture (PMOF) and hip fracture (PHF) were calculated using the FRAX tool. based on the individuals’ information collected from the questionnaire. Taking into account the study’s primary focus on lung cancer screening and the concern regarding additional radiation exposure in this cohort, we opted not to evaluate femoral neck BMD. Consequently, the FRAX tool without femoral neck BMD input was employed to evaluate PMOF and PHF.

### Fracture assessment

Incident fractures were self-reported, and VFs were assessed in all T1-L2 vertebrae on sagittal CT images at the 3-year follow-up using the Genant semi-quantitative criteria (18). The grade of each VF was independently assessed by two radiologists through visual inspection, comparing the height or area of the affected vertebra with that of the adjacent superior and inferior vertebral bodies, and classified as normal (Grade 0), mild (Grade 1, approximately 20-25% reduction in any height and/or 10-20% reduction in area), moderate (Grade 2, approximately 25-40% reduction in any height and/or 20-40% reduction in area), and severe (Grade 3, approximately >40% reduction in any height and/or area). Any discrepancies between the two radiologists were resolved by consensus.

### Statistical analysis

Statistical analyses were conducted utilizing SPSS(version 25.0; IBM Corp, Armonk, NY, USA) and MedCalc (version 18.2.1; MedCalc Software, Ostend, Belgium). Categorical variables were presented as frequencies and percentages, whereas continuous variables with skewed distributions were expressed as medians and interquartile ranges (IQRs).

Interobserver agreement for VF grading, assessed according to the Genant semi-quantitative method, was evaluated using the weighted kappa statistic to ensure reliability between the two radiologists. Participants were stratified into normal BMD, osteopenia, and osteoporosis groups according to LBMD status. Differences among groups were analyzed using the Kruskal–Wallis test for continuous variables and either Pearson’s chi-square test or Fisher’s exact test for categorical variables, as appropriate.

Logistic regression analyses were employed to assess the associations between baseline TBMD, LBMD, and FRAX estimates and CT-detected VFs or self-reported incident fractures. Variables with a p-value <0.1 in univariate analyses were included in the multivariable models. Odds ratios (ORs) and corresponding confidence intervals (CIs) were reported. Receiver operating characteristic (ROC) curve analyses were used to determine the optimal BMD cutoff values for fracture outcomes, and the corresponding sensitivity and specificity were reported. Six binary logistic regression models were established to evaluate the performance of different bone health metrics for CT-detected VFs. Model 1 included TBMD alone, and Model 2 included LBMD alone. Model 3 combined TBMD with current smoking status, while Model 4 combined LBMD with current smoking status. Model 5 was based on the FRAX-derived PMOF, and Model 6 was based on the FRAX-derived PHF. Comparisons of the areas under the ROC curves (AUCs) were performed using DeLong’s test. A two-sided p-value <0.05 was considered statistically significant.

## Results

### Characteristics of participants

The basic characteristics of the participants, categorized by LBMD status, are presented in **Table 2**. The final cohort consisted of 546 individuals (297 females and 249 males) with a median age of 62 years (IQR: 57.0 - 65.0 years). The median TBMD and LBMD were 115.3 mg/cm³ (IQR: 93.8-140.0 mg/cm³) and 102.3 mg/cm³ (IQR: 83.3 -127.0 mg/cm³), respectively. Among the participants, 46.9% (n=256) were diagnosed with osteopenia, while 22.2% (n=121) were diagnosed with osteoporosis. During a median follow-up of 36.4 months (Range: 34.8-42.4 months), 16 participants (2.9%) reported incident fractures. VFs were identified in 323 participants on follow-up CT scans, of which 21.2% (n=116) classified as moderate (Grade 2) or severe (Grade 3). Interobserver agreement for VF classification between the two radiologists was 77.5%, with a weighted kappa value of 0.648 (**Supplementary Table 1**).

**Table 2:** The characteristics of participants by LBMD groups.

Variables	Total(*n* = 546)	Normal BMD (*n* = 169)	Osteopenia(*n* = 256)	Osteoporosis (*n* = 121)	*P*
Baseline					
Age	62.0 (57.0, 65.0)	58.0 (53.0, 63.0)	62.0 (59.0, 65.0)	65.0 (62.0, 68.0)	**<0.001**
Sex					0.766
Men	249 (45.6%)	81 (47.9%)	114 (44.5%)	54 (44.6%)	——
Women	297 (54.4%)	88 (52.1%)	142 (55.5%)	67 (55.4%)	——
Height	165.0 (160.0, 172.0)	165.0 (160.0, 172.0)	165.0 (159.0, 172.0)	164.0 (160.0, 172.0)	0.510
Weight	67.3 (60.0, 75.0)	68.0 (60.0, 75.0)	68.0 (60.0, 75.0)	65.0 (60.0, 74.0)	0.498
Current smoking	115 (21.1%)	30 (17.8%)	55 (21.5%)	30 (24.8%)	0.340
Excessive alcohol consumption	53 (9.7%)	18 (10.7%)	22 (8.6%)	13 (10.7%)	0.711
Secondary osteoporosis	100 (18.3%)	25 (14.8%)	49 (19.1%)	26 (21.5%)	0.312
Glucocorticoids	11 (2.0%)	3 (1.8%)	6 (2.3%)	2 (1.7%)	1.000
Parental hip fracture	41 (7.5%)	9 (5.3%)	19 (7.4%)	13 (10.7%)	0.225
Previous fragility fracture	21 (3.8%)	2 (1.2%)	4 (1.6%)	15 (12.4%)	**<0.001**
FRAX					
PMOF (%)	2.9 (2.2, 4.0)	2.3 (1.9, 3.2)	3.0 (2.2, 4.0)	3.8 (2.7, 4.9)	**<0.001**
PHF (%)	0.7 (0.4, 1.2)	0.5 (0.2, 0.8)	0.6 (0.4, 1.2)	1.1 (0.7, 1.6)	**<0.001**
TBMD (mg/cm^3^)	115.3 (93.8, 140.0)	151.1 (142.0, 168.4)	111.7 (100.7, 122.1)	77.8 (69.6, 86.0)	**<0.001**
LBMD (mg/cm^3^)	102.3 (83.3, 127.0)	136.6 (128.5, 152.9)	98.0 (88.3, 109.3)	66.9 (59.0, 73.7)	**<0.001**
3-year follow-up					
TBMD* (mg/cm^3^)	108.2 (87.1, 132.3)	142.1 (132.3, 160.0)	104.5 (93.6, 115.1)	72.5 (63.4, 79.5)	**<0.001**
LBMD* (mg/cm^3^)	94.9 (76.2, 118.4)	129.4 (118.8, 147.5)	92.3 (82.5, 103.8)	61.5 (53.2, 70.0)	**<0.001**
Self-reported incident fracture	16 (2.9%)	3 (1.8%)	6 (2.3%)	7 (5.8%)	0.123
VF	323 (59.2%)	40 (23.7%)	170 (66.4%)	113 (93.4%)	**<0.001**
Grade 1	207 (37.9%)	32 (18.9%)	114 (44.5%)	61 (50.4%)	——
Grade 2	101 (18.5%)	8 (4.7%)	48 (18.8%)	45 (37.2%)	——
Grade 3	15 (2.7%)	0 (0.0%)	8 (3.1%)	7 (5.8%)	——

Statistically significant values are identified in boldface.

As shown in **Table 2**, significant differences were observed among the normal BMD, osteopenia, and osteoporosis groups in terms of distribution of age, previous fragility fracture, FRAX scores, BMD values, and VF prevalence (all p < 0.001). Participants with osteoporosis were older and had higher rates of previous fragility fracture (12.4% vs. 1.6% vs. 1.2%), higher FRAX estimates of PMOF (3.8% [2.7–4.9] vs. 3.0% [2.2–4.0] vs. 2.3% [1.9–3.2]) and PHF (1.1% [0.7–1.6] vs. 0.6% [0.4–1.2] vs. 0.5% [0.2–0.8]), lower TBMD (77.8 [69.6–86.0] vs. 111.7 [100.7–122.1] vs. 151.1 [142.0–168.4] mg/cm³) and LBMD (66.9 [59.0–73.7] vs. 98.0 [88.3–109.3] vs. 136.6 [128.5–152.9] mg/cm³), higher VF prevalence (93.4% vs. 66.4% vs. 23.7%) compared with osteopenia and normal BMD.

### Association of TBMD, LBMD and FRAX with CT-detected VF

**Table 3** provides the results of the binary logistic regression analyses for CT-detected VF. Statistically significant differences were observed between participants with and without CT-detected VF in age and current smoking, TBMD, LBMD, PMOF, PHF. CT-detected VF was significantly associated with TBMD and LBMD independent of adjustment for age and current smoking. In the unadjusted binary logistic regression analyses, ORs were 0.955 (95% CI: 0.947 – 0.963) for TBMD and 0.952 (95% CI: 0.944 – 0.961) for LBMD. Adjustment for age and current smoking did not significantly change the associations, with adjusted ORs of 0.953 (95% CI: 0.944–0.962) for TBMD and 0.950 (95% CI: 0.941–0.959) for LBMD.

**Table 3:** Binary logistic regression analysis to CT-detected VF.

Variables	Univariate analysis		Multivariate analysis of TBMD		Multivariate analysis of LBMD	
	Crude OR (95% cl)	*P*	Adjusted OR (95% cl)	*P*	Adjusted OR (95% cl)	*P*
Age	1.069 (1.040, 1.099)	**<0.001**	0.979 (0.945, 1.015)	0.247	0.975 (0.940, 1.011)	0.169
Sex	0.790 (0.560, 1.114)	0.179				
Height	0.997 (0.975, 1.020)	0.813				
Weight	0.999 (0.984, 1.014)	0.889				
Current smoking	2.064 (1.316, 3.237)	**0.002**	2.365 (1.370, 4.085)	**0.002**	2.327 (1.342, 4.033)	**0.003**
Excessive alcohol consumption	1.263 (0.701, 2.277)	0.437				
Glucocorticoids	0.825 (0.249, 2.738)	0.754				
Secondary osteoporosis	1.284 (1.819, 2.012)	0.276				
Parent fractured hip	1.533 (0.776, 3.030)	0.219				
Previous fragility fracture	2.272 (0.820, 6.296)	0.114				
TBMD (mg/cm^3^)	0.955 (0.947, 0.963)	**<0.001**	0.953 (0.944, 0.962)	**<0.001**		
LBMD (mg/cm^3^)	0.952 (0.944, 0.961)	**<0.001**			0.950 (0.941, 0.959)	**<0.001**
FRAX						
PMOF (%)	1.149 (1.039, 1.270)	**0.007**				
PHF (%)	1.433 (1.139, 1.804)	**0.002**				

Statistically significant values are identified in boldface. P<0.05.

**Figure 1:**
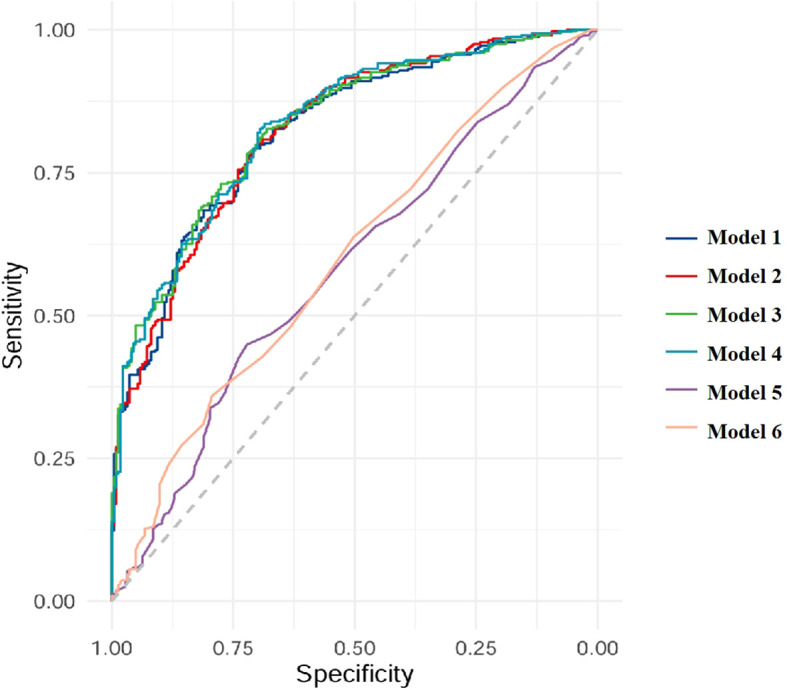
Receiver operating characteristics curves for CT-detected VFs.

The models of TBMD, LBMD and FRAX for CT-detected VF were implemented using binary logistic regression analyses. In total, six models (Models 1–6) were developed based on different combinations of these variables, as described in the Methods section. AUC analyses indicated that all models were capable of identifying participants with VFs (**Table 4**, **Figure 1**). When using BMD alone, the AUC values were 0.823 (95% CI: 0.788–0.858) for TBMD-based Model 1 and 0.824 (95%CI: 0.790–0.859) for LBMD-based Model 2. After the inclusion of BMD and current smoking in the models, the corresponding AUCs for Model 3 and Model 4 were 0.832 (95% CI 0.799–0.866) and 0.833 (95% CI: 0.800–0.860), respectively. In contrast, the AUCs for PMOF-based Model 5 and PHF-based Model 6 were substantially lower at 0.582 (95% CI 0.534–0.631) and 0.599 (95% CI: 0.551–0.647), respectively.

**Table 4:** Receiver operating characteristic analysis of CT-detected VFs.

Models	AUC (95% CI)	*p*	sensitivity	specificity
Model 1	0.823 (0.788, 0.858)	**<0.001**	0.793	0.709
Model 2	0.824 (0.790, 0.859)	**<0.001**	0.802	0.700
Model 3	0.832 (0.799, 0.866)	**<0.001**	0.824	0.682
Model 4	0.833 (0.800, 0.860)	**<0.001**	0.836	0.686
Model 5	0.582 (0.534, 0.631)	**0.001**	0.449	0.722
Model 6	0.599 (0.551, 0.647)	**<0.001**	0.359	0.794

Model 1 and Model 2 only included TBMD and LBMD, respectively. Model 3 incorporated TBMD and current smoking. Model 4 incorporated LBMD and current smoking. Model 5 and Model 6 only used PMOF and PHF, respectively. Statistically significant values are identified in boldface.

The BMD-based models (Model 1 - 4) demonstrated significantly higher discriminatory performance compared with the FRAX-based Model 5 and Model 6 without BMD input (all *p* < 0.001), however, no significant difference was observed between TBMD-based Model 1 and LBMD-based Model 2 (*p* = 0.752, **Figure 1**, **Table 5**). Furthermore, the incorporation of current smoking status in Model 3 did not yield a statistically significant improvement compared with Model 1 (*p* = 0.058). Based on ROC analysis, the optimal cutoff of TBMD for CT-detected VF was 124 mg/cm^3^, which achieved a sensitivity of 79.3% and a specificity of 70.9% (**Table 4**).

**Table 5:** Comparison of models (DeLong test).

Models	Model 2	Model 3	Model 4	Model 5	Model 6
Model 1	0.752	0.058	0.083	**<0.001**	**<0.001**
Model 2		0.169	**0.047**	**<0.001**	**<0.001**
Model 3			0.759	**<0.001**	**<0.001**
Model 4				**<0.001**	**<0.001**
Model 5					0.235

Statistically significant values are identified in boldface.

### Association between TBMD, LBMD, FRAX and self-reported incident fracture

**Table 6** presents the results of binary logistic regression analysis to self-reported incident fracture. Participants with self-reported incident fractures showed statistically significant differences in excessive alcohol consumption and TBMD when compared to those without self-reported incident fractures. Notably, TBMD was significantly associated with self-reported incident fractures, independent of adjustment for excessive alcohol consumption. The unadjusted binary logistic regression analysis yielded an OR of 0.982 (95% CI: 0.965–0.999) for TBMD, and this association remained unchanged after adjustment for excessive alcohol consumption (OR: 0.982, 95% CI: 0.965–0.999). In contrast, LBMD, PMOF and PHF did not demonstrate significant associations with self-reported incident fractures (all *p* > 0.05).

**Table 6:** Binary logistic regression analysis to self-reported incident fractures.

Variables	Univariate analysis		Multivariate analysis of TBMD			Multivariate analysis of LBMD	
	Crud OR (95%cl)	*P*	Adjusted OR (95%cl)		*P*	Adjusted OR (95%cl)	*P*
Age	0.999 (0.926, 1.079)	0.987					
Sex	1.411 (0.506, 3,939)	0.511					
Height	0.960 (0.898, 1.026)	0.232					
Weight	0.962 (0.916, 1.010)	0.117					
Current smoking	0.861 (0.241, 3.075)	0.818					
Excessive alcohol consumption	3.272 (1.016, 10.534)	**0.047**	3.297 (1.014, 10.722)		**0.047**	3.180 (0.980, 10.314)	0.054
Glucocorticoids	3.467 (0.417, 28.845)	0.250					
Secondary osteoporosis	1.507 (0.476, 4.773)	0.486					
Parent fractured hip	0.817 (0.105, 6.342)	0.846					
Previous fragility fracture	1.700 (0.214, 13.512)	0.616					
TBMD (mg/cm^3^)	0.982 (0.965, 0.999)	**0.041**	0.982 (0.965,0.999)		**0.041**		
LBMD (mg/cm^3^)	0.984 (0.967, 1.001)	0.072				0.984 (0.967, 1.002)	0.077
FRAX							
PMOF (%)	1.038 (0.817, 1.321)	0.758					
PHF (%)	1.146 (0.767, 1.715)	0.506					

Statistically significant values are identified in boldface.

**Figure 2:**
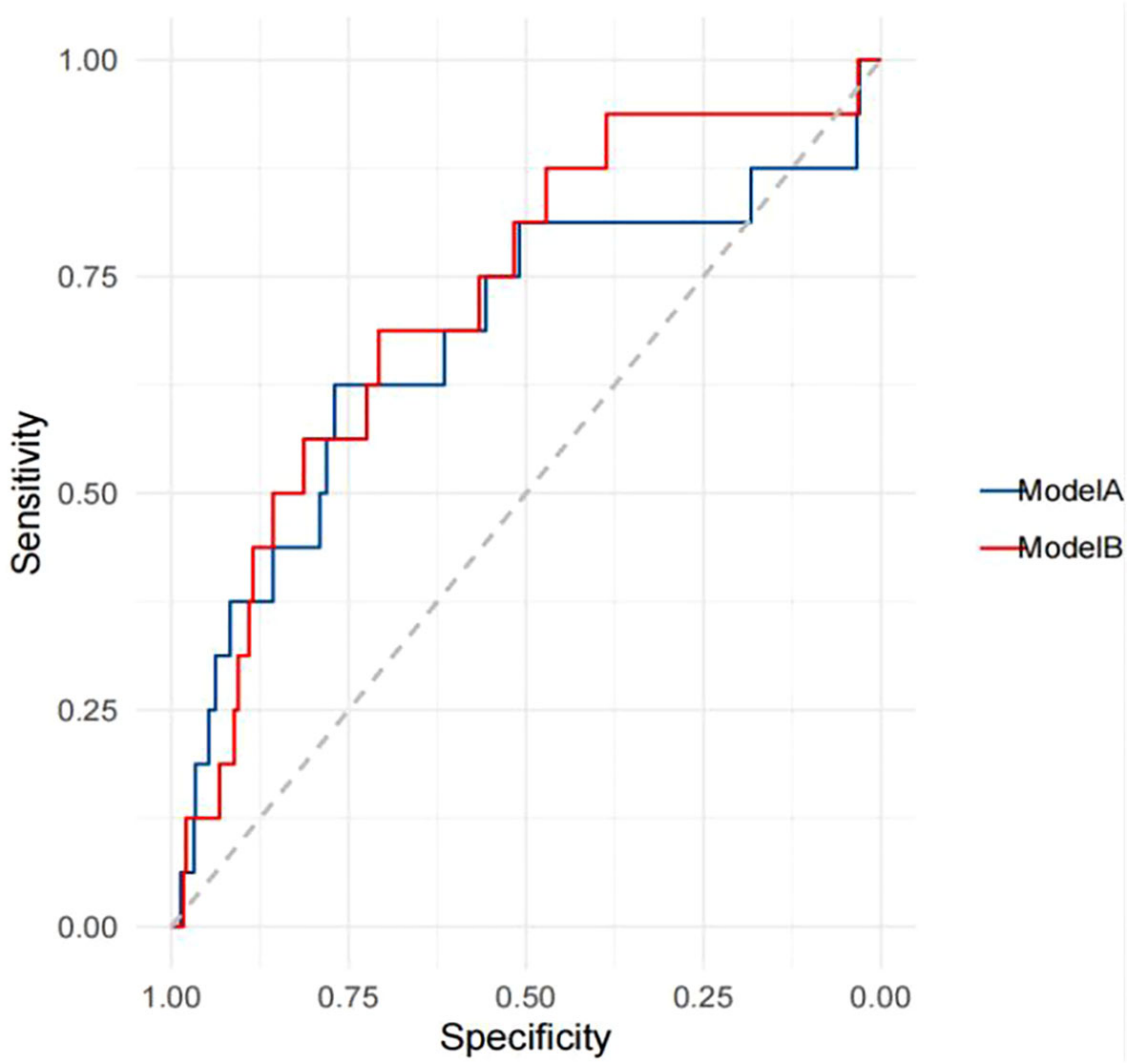
Receiver operating characteristics curves for the prediction of self-reported incident fractures.

The performance of TBMD-based predictive models for self-reported incident fractures is presented in **Table 7**. The AUC for predicting self-reported incident fractures using TBMD alone (Model A) was 0.678 (95% CI: 0.520 - 0.837). Incorporating TBMD and excessive alcohol consumption in model (Model B) did not significantly improve predictive performance for self-reported incident fractures compared with TBMD only (AUC = 0.723 vs. 0.678, *p* = 0.369, **Figure 2**, **Table 7**).

**Table 7:** Receiver operating characteristic analysis of self-reported incident fractures.

Models	AUC (95%CI)	*P*	Sensitivity	Specificity
Model A	0.678 (0.520, 0.837)	**<0.001**	0.625	0.770
Model B	0.723 (0.595, 0.850)	**<0.001**	0.688	0.708

Model A only included TBMD. Model B included TBMD and excessive alcohol consumption. Statistically significant values are identified in boldface.

## Discussion

Our study demonstrated that lower QCT-based TBMD is associated with higher VF and incident fracture rates in lung cancer screening populations in China. The findings indicated that TBMD performs as well as LBMD in identifying participants with VF and also aids in assessing the risk of incident fracture. Although TBMD and LBMD demonstrated comparable discriminatory performance, the primary advantage of TBMD lies in its opportunistic use from routinely acquired chest LDCT scans rather than superior accuracy. This characteristic enables bone health evaluation without additional imaging, radiation exposure, or cost, underscoring TBMD’s potential clinical utility for large-scale population screening. This discovery addresses two critical gaps in current screening paradigms: the unmet need for bone health assessment in people over 40 years old, and the anatomical limitations of standard LDCT protocols.

Existing evidence indicates that QCT combined with chest LDCT for measuring LBMD is feasible for opportunistic screening osteoporosis and reported a 13.5% prevalence of osteoporosis among man and a 29.0% prevalence among women in Chinese aged ≥50 years (13, 19). In this lung cancer screening populations aged over 40 years, the prevalence of osteoporosis is 22.2% (21.7% among man and 22.6% among woman). Notably, the prevalence of VF (59.2%) observed in this cohort exceeds that reported in most previous studies (10–65%) (2, 5, 7, 10, 20, 21). This may be partly attributable to the characteristics of our study population, as participants voluntarily undergoing opportunistic BMD assessment during chest LDCT screening may be more prone to bone health issues. Additionally, the prevalence of smoking in this cohort is comparatively elevated (22), particularly among men with 69.1% ever smokers comprising 25.7% former smokers and 43.4% current smokers (**Supplementary Table 2**). This also supported the higher osteoporosis prevalence in men (21.7% vs. 5.0% in prior population-based reports) (13). Collectively, these factors provide a plausible explanation for the higher VF prevalence observed in our study. Over a 3-year follow-up period, 2.9% of participants had self-reported incident fractures. In contrast, the prevalences of clinical fracture were around 4.1% over 5 years and 7.6% over 10 years (2, 23).

Current research on assessing fracture risk using QCT-based BMD primarily focuses on utilizing LBMD (5, 7, 20). However, the China Health Big Data project underscores the limitations of current diagnostic paradigms that rely solely on lumbar BMD. We focused on using TBMD as an alternative to LBMD for estimating the risk of fracture in lung cancer screening populations, suggesting TBMD thresholds for prevalent VF and incident fracture. In our study, TBMD demonstrated comparable efficacy to LBMD in estimating prevalent VF with AUC values of 0.823 and 0.824, respectively (*p* = 0.752). This finding aligns with the conclusions of a previous study, which reported AUC values of 0.72 for both TBMD and LBMD (10). We identified a TBMD threshold of 124 mg/cm^3^ for VF, achieving a sensitivity of 79.3% and a specificity of 70.9%. However, the study conducted by Ramschütz et al. did not determine an optimal TBMD cutoff value specifically for VF (10). We further found that TBMD is significantly associated with self-reported incident fractures and established a threshold of 94 mg/cm^3^, with a sensitivity of 62.5%, a specificity of 77.0% and AUC value of 0.678. Some studies have shown that TBMD derived from cardiac CT is useful for identifying individuals with a high risk of incident fractures (11, 24, 25). Among them, Therkildsen et al. established a TBMD cutoff value for optimal prediction of any incident fracture at 102.6 mg/cm^3^, with a sensitivity of 54%, a specificity of 66% and a corresponding AUC of 0.60 (24). In comparison, our threshold is lower but its corresponding sensitivity, specificity and AUC values are higher. The lower threshold of TBMD in our study is likely attributable to the fact that our thoracic spine levels (T11-T12) are positioned inferiorly compared with those examined in their study, which focused on three contiguous vertebrae at and below the level of origin of the left main coronary artery. Hu et al. determined a threshold value of 91 mg/cm³ for TBMD at the T11-T12 vertebrae to diagnose osteoporosis in a lung cancer screening population. They asserted that osteoporosis identified using the threshold is associated with incident fractures. However, to the best of our knowledge, they did not provide comprehensive evidence regarding the performance of this threshold in predicting fracture risk, nor did they compare its capability with that of LBMD.

The majority of prior studies on opportunistic BMD measurements has focused on patients undergoing routine CT scans, many of whom present with specific comorbidities such as cancer (20, 26). Additionally, there is currently a lack of clarity regarding which populations should be targeted for opportunistic screening. Our study was population-based and conducted within the context of lung cancer screening. Over the past decade, the use of LDCT for lung cancer screening has progressively increased, offering an opportunity for the opportunistic assessment of TBMD without incurring additional radiation exposure, cost, or extended scanning time. Advanced age and smoking are well-established risk factors for both fractures and lung cancer (27, 28). Chinese guidelines have extended the recommended age for lung cancer screening to individuals over 40 years old (29), a demographic characterized by a high prevalence of osteoporosis and fractures (2). Moreover, a study revealed that lung cancer screening participants have a substantial VF burden, particularly among current smokers (30). Our study corroborates this finding, identifying current smoking as an independent risk factor for VF, with an odds ratio of 2.365 (95% CI: 1.370 – 4.085). These results indicated that shared risk factors should be carefully considered in combined screening for lung cancer and BMD, which may help standardize the target populations that should undergo opportunistic screening. Our study is among the first to report that LDCT-based TBMD is effective in identifying individuals with prevalent VF and a high risk of incident fracture in lung cancer screening populations, with similar effectiveness to LBMD. These findings imply that opportunistic TBMD could serve as a viable alternative for estimating the risk of prevalent VF and any incident fracture when LBMD is unavailable.

There are limitations to this prospective study. Firstly, this is a single-center study, and the study population is limited to community residents aged 40–74 who participated in lung cancer screening. However, this is exactly the population that could benefit from opportunistic BMD screening because LDCT scans already exist. Secondly, although our study is population-based, the purpose of screening for lung cancer may lead to bias of people at high risk of lung cancer. Moreover, our study includes individuals undergoing chest LDCT for lung cancer screening, aiming to utilize simpler predictors for easy clinical integration without extra radiation. Therefore, FRAX was applied without femoral neck BMD input, which likely reduced its predictive performance. The comparison between TBMD and FRAX in this study therefore reflects the relative performance of TBMD versus FRAX without BMD. FRAX models incorporating femoral neck BMD, where available, would be expected to provide higher accuracy. Consequently, the observed superiority of TBMD over FRAX should be interpreted with caution. In addition, the occurrence of incident fractures was self-reported via questionnaires among the participants who attended the follow-up. Thus, the participants who experienced severe fractures might not have attended the follow-up, potentially leading to an underestimation of fracture incidence. Finally, it should be noted that the low incidence of self-reported fractures (2.9%) limits the ability to evaluate the prospective predictive value of TBMD for future fracture events. Future studies with larger sample sizes and longer follow-up periods are warranted to validate the predictive value of TBMD in fracture risk assessment.

In conclusion, this study suggests that QCT-based TBMD opportunistically derived from routine chest LDCT scans may help identify individuals with vertebral fractures and an elevated risk of incident fracture in lung cancer screening populations. With performance comparable to LBMD and no need for additional imaging or radiation, TBMD shows promise as a practical tool for opportunistic bone health assessment, though further validation in larger, long-term studies is needed.

## Data Availability

The original contributions presented in the study are included in the article/**supplementary material**. Further inquiries can be directed to the corresponding authors.
