# Supplementary material for: Thoracic vertebral bone mineral density measured by quantitative computed tomography is associated with fracture risk in lung cancer screening populations: a prospective cohort study
Source: Front Endocrinol (Lausanne). 2025 Nov 13;16:1672551. doi: 10.3389/fendo.2025.1672551 (PMC12657172; doi:10.3389/fendo.2025.1672551)
Supplement: Supplementary file 1 [file Table1.docx]

**Supplemental Table 1** Agreement between the two radiologists for VF classification

|  | | **VF-1** | | | | **Total** | **Kappa value** |
| --- | --- | --- | --- | --- | --- | --- | --- |
|  |  | **Grade 0** | **Grade 1** | **Grade 2** | **Grade 3** |  |  |
| VF-2 | Grade 0 | 176 | 40 | 0 | 0 | 216 | 0.648 |
|  | Grade 1 | 27 | 182 | 2 | 0 | 211 |  |
|  | Grade 2 | 3 | 44 | 56 | 1 | 104 |  |
|  | Grade 3 | 0 | 0 | 6 | 9 | 15 |  |
| Total | | 206 | 266 | 64 | 10 | 546 |  |

VF. Vertebral fracture
