# Supplementary material for: Thoracic vertebral bone mineral density measured by quantitative computed tomography is associated with fracture risk in lung cancer screening populations: a prospective cohort study
Source: Front Endocrinol (Lausanne). 2025 Nov 13;16:1672551. doi: 10.3389/fendo.2025.1672551 (PMC12657172; doi:10.3389/fendo.2025.1672551)
Supplement: Supplementary file 2 [file Table2.docx]

**Supplementary Table 2** Smoking status of the participants

|  | **Total (n=546)** | **Men (n=249)** | **Women (n=297)** |
| --- | --- | --- | --- |
| Ever smoking | 181 (33.2%) | 172 (69.1%) | 9 (3.0%) |
| Former smoking | 66 (12.1%) | 64 (25.7%) | 2 (0.7%) |
| Current smoking | 115 (21.1%) | 108 (43.4%) | 7 (2.4%) |
